# A Strange Occurrence of Hematohidrosis: A Case Report From Saudi Arabia

**DOI:** 10.7759/cureus.21682

**Published:** 2022-01-28

**Authors:** Sara Alasfoor, Muna Albashari, Aya Alsermani, Mohamad Bakir, Maamoun Alsermani, Sami Almustanyir

**Affiliations:** 1 College of Medicine, Alfaisal University, Riyadh, SAU; 2 College of Medicine, Dar Al Uloom University, Riyadh, SAU; 3 Hematology, Prince Mohammed Bin Abdulaziz Hospital, Riyadh, SAU; 4 Research, Ministry of Health, Riyadh, SAU

**Keywords:** hematidrosis, hematohidrosis, bloody sweat, stress, beta-blocker, propranolol, inderal, case report

## Abstract

Hematohidrosis is a rare disorder of blood excretion from the eccrine sweat gland not associated with an injury or trauma. Although several hypotheses exist to explain such a condition, the etiology of hematohidrosis remains unknown. Psychological stress is strongly linked to the condition, yet patients may present completely healthy with no identifiable etiology. The diagnosis of hematohidrosis can be difficult and requires the exclusion of bleeding disorders, vasculitis, and other disorders. Here, we present a case of hematohidrosis in a 20-year-old female who had almost weekly bloody tears, as well as gum bleeding, ear bleeding, and epistaxis for the past four months. During hospitalization, the patient was thoroughly investigated for an etiology, but no identifiable cause was found. The patient was diagnosed with hematohidrosis and treated with propranolol. Her condition had improved significantly on follow-up.

## Introduction

Hematohidrosis is a disorder of the sweat glands characterized by bloody sweat [1]. Patients’ bodies tend to excrete blood from the skin and mucous membranes (forehead, palm, ear, etc.), which is unrelated to a skin injury or trauma. It is an extremely rare condition with only a few cases reported in the literature. The epidemiological distribution is primarily in Asia and Africa [2]. In many reported cases, acute fear and chronic stress or anxiety have been linked to the development of the disease. On the other hand, some cases developed in otherwise healthy patients with no clear cause [2]. In Saudi Arabia, only one case of a nine-year-old girl with no prior psychological or clinical disorders has been reported [3]. Here, we present the case of a 20-year-old female with hematohidrosis for the past four months.

## Case presentation

A 20-year-old Saudi female presented with almost weekly bleeding from both eyes, which she described as “bloody tears.” She also reported gum bleeding, mild ear bleeding, and epistaxis for the previous four months. Her medical history was significant for retinoblastoma of the left eye at the age of nine years, which resulted in enucleation of the left eye and artificial eye placement. She also had a history of prolonged and heavy menstrual cycles for a few months, fibroadenoma for six months, and neurological symptoms, including leg weakness with normal lab investigations. She was on escitalopram for her complaints. Moreover, she was diagnosed with sinus tachycardia using a Holter monitor. The patient’s family history was unremarkable except for her uncle who was known to have an anxiety disorder. On physical examination, she had eczema, joint pain, and scoliosis. She had no bruising, splenomegaly, lymphadenopathy, synovitis, or oral ulcers. The results of the lab tests, which included a complete blood count, coagulation profile, fibrinogen, thyroid function test, as well as antinuclear antibodies, extractable nuclear antigen, and immunoglobulin E, were all normal, excluding the presence of vasculitis or rheumatic diseases. However, the patient had a slightly high red blood cell count and a mildly low vitamin B12 level. The patient was diagnosed with hematohidrosis and prescribed propranolol (Inderal) 20 mg twice daily which improved her condition significantly. The patient was followed up twice in the past seven months. On the first visit within three months, she reported having five less severe episodes. On the second visit within four months, she reported having only one episode.

## Discussion

Hematohidrosis is defined as spontaneous and temporary blood excretion from eccrine sweat glands [3]. The pathophysiological theories proposed for the condition include high vascular pressure causing blood to pass into the ducts of sweat glands, dermal vessels vasculitis, and sympathetic nervous system activation, which causes the periglandular vessel to constrict and then expand, allowing blood to pass to the duct and causing hematohidrosis [4]. The typical presentation of the clinical phenomena is young girls (between the ages of nine and fifteen years) with a positive history of psychological problems presenting with bleeding from an intact skin that lasts for a short time and resolves on its own or after a course of propranolol, psychotherapy, or both [3]. Two distinguishing features can be used to identify the disease as a distinctive entity. The first is that laboratory and clinical investigations of people with hematohidrosis are usually normal, excluding blood disorders, chromhidrosis, or self-inflicted skin lesions. The second is that most patients improve or go into remission after taking beta-blockers and/or antidepressants, antianxiolytics, or psychotherapy [2]. According to a literature review done in 2021, treatment with beta-blockers had an effectiveness of up to 94%, while psychotherapy (including cognitive-behavioral therapy, relaxation techniques, and family education) had an effectiveness of 66% [5]. Idiosyncratically, our patient was a 20-year-old female, unlike the usual age presentation of the disease. It has been reported that 83% of hematohidrosis patients present at age 18 and younger [5]. Although our case involved a Saudi female, hematohidrosis has been rarely reported in the region. Out of 36 multinational cases of hematohidrosis, the highest number of cases were in India with a total of 13 patients, followed by South Africa with four, and Iran with three [5]. Although psychological stress is strongly linked to the disorder, our patient did not report psychological problems other than a second-degree family history of anxiety disorder [1]. Furthermore, four cases in the literature had a significant non-psychiatric history prior to developing the clinical disorder, one of which was a known case of epilepsy and the other three cases had a head injury. All of these cases provide insights into the underlying etiology of the disease. Our patient had a history of retinoblastoma as well as neurological symptoms, suggesting that hematohidrosis may be linked to neurological pathology disorders [2,5,6]. However, no definitive etiology has been established.

## Conclusions

Hematohidrosis can be a frightening experience for the patient and their family and often leads to extensive unnecessary testing. Reporting these rare cases can aid in educating physicians globally about this benign yet scary condition as well as ease the management. Physicians should suspect hematohidrosis whenever a patient exhibits similar symptoms in the absence of an underlying disease, even if the patient does not have any psychological disorders or stressors. Although it is a rare condition, early detection and treatment are associated with favorable outcomes and complete remission of the disease.
